# Seroprevalence and risk factors associated with *Leptospira* Hardjo among commercial dairy cattle farms of Rupandehi district, Nepal

**DOI:** 10.1186/s12917-025-04882-x

**Published:** 2025-07-05

**Authors:** Tulsi Ram Gompo, Sudiksha Pandit, Deepak Subedi, Ram Chandra Sapkota, Aditi Pandey, Rojina Nepal, Ananda Tiwari, Sumit Jyoti

**Affiliations:** 1 Central Veterinary Laboratory, Department of Livestock Services, Tripureswor, Kathmandu, 46000 Nepal; 2 Melbourne Veterinary School, University of Melbourne, Parkville, Melbourne, Victoria 3010 Australia; 3 Paklihawa Campus, Institute of Agriculture and Animal Science, Tribhuvan University, Paklihawa, Nepal; 4 Department of Veterinary Microbiology and Pathology, College of Veterinary Medicine, Washington State University, Pullman, WA 99164 USA; 5 Department of Poultry Science, University of Georgia, Athens, Georgia 30602 USA; 6 Department of Food Hygiene and Environmental Health, Faculty of Veterinary Medicine, University of Helsinki, Helsinki, 00014 Finland; 7 Department of Health Management, Atlantic Veterinary College, University of Prince Edward Island, Charlottetown, PEI C1A 4P3 Canada

**Keywords:** Leptospirosis, Dairy cattle, Zoonoses, Nepal, Risk factors, Occupational hazard, Hardjo

## Abstract

**Background:**

Nepal relies on an agrarian-based economy, with the livestock sector contributing significantly to the national GDP. However, diseases like leptospirosis negatively impact cattle production and pose significant zoonotic risks. This study represents the first attempt to evaluate the risk factors of leptospirosis in cattle in Nepal. A cross-sectional study was conducted from March 2019 to April 2020 in 14 administrative units of the Rupandehi district. A total of 367 blood samples were collected from 206 cattle farms using a proportionate sampling procedure. An indirect ELISA was used to detect specific antibodies in serum samples against *Leptospira interrogans* serovar Hardjo. Farm management practices and knowledge of zoonotic diseases were assessed through interviews with animal owners from the 206 cattle farms. Regression analyses were conducted to analyze the herd and farm level risk factors.

**Results:**

The overall farm-level seroprevalence of leptospirosis was 4.85% (95% CI: 2.35–8.75), while the animal-level seroprevalence was 3.81% (95% CI: 2.10–6.30). Using multivariable logistic regression analysis, we found that farms with purchased cattle (farms that regularly introduce cattle from other farms) had a borderline significant increase in odds of leptospirosis (OR: 7.25, 95% CI: 0.88–59.46, *p* = 0.065) compared to farms that only keep home-bred cattle. Additionally, larger farms (> 10 animals) were significantly associated with increased odds of leptospirosis (OR: 13.34, 95% CI: 1.64–108.42, *p* = 0.015) compared to smaller farms (≤ 10 animals). At the animal level, no statistically significant difference was observed in the multivariable mixed-effects logistic regression model, which included farm as a random effect.

**Conclusion:**

The detection of farms with positive serum samples highlights the persistent threat of leptospirosis to cattle production and its occupational hazards within Nepal’s dairy sector. Farm-level risk factors, such farms with purchased cattle and larger farm sizes, emphasize the need for targeted control measures. Given the zoonotic nature of the disease and its ecological complexity involving multiple hosts, a One Health approach is essential. Collaborative efforts among stakeholders are needed to develop evidence-based policies, strengthen health system preparedness, and implement practical interventions that reduce transmission risks and the overall disease burden in both human and animal populations across the country.

**Supplementary Information:**

The online version contains supplementary material available at 10.1186/s12917-025-04882-x.

## Background

Leptospirosis is a bacterial zoonosis caused by the infection with *Leptospira* spp., leading to both clinical and subclinical manifestations in humans and animals [1]. The natural carriers of this bacteria are wild and domestic animals, rodents, and dogs [2]. Among the various serovars, *Leptospira interrogans* serovar Hardjo and *Leptospira borgpetersenii* serovar Hardjo (collectively referred to as *L.* Hardjo) are commonly found in cattle, which serve as the primary maintenance host for these serovars [3]. The bacteria are shed in both the urine and discharge from the genitalia of the infected cattle [4]. In cattle, leptospirosis causes reduced conception rates and reproductive problems such as abortions, stillbirths, mummifications, the birth of weak calves, and decreased milk production [4]. The principal risk factors identified for seropositivity of leptospirosis among cattle are cattle with a communal grazing practice [5], use of surface drinking water sources [6], access of dogs to pastures, contact of rodents with feed sources [7], and the introduction of new animals into the farm [8].

Humans are at risk of contracting leptospirosis through various sources, including contact with floodwaters, exposure to infected livestock, direct contact of wounds with contaminated soil during work, and exposure to soil or water contaminated with rat urine [3, 9]. The occupational risk of exposure is mainly among veterinarians, farmers, abattoir workers, hunters, animal shelter workers, and agricultural workers [3]. The World Health Organization (WHO) has considered leptospirosis a neglected and re-emerging zoonotic disease for humans and animals [10]. According to WHO, over 500,000 cases of leptospirosis are reported worldwide each year [10]. Still, many cases are missing due to a lack disease awareness and appropriate diagnostic facilities [11]. It is also estimated that around 1.03 million cases are detected every year, and the yearly mortality goes up to 58,900 worldwide [12].

One-third of Nepal’s economy is based on agriculture, and the livestock sector alone contributes around 12% to the national GDP [13]. Cattle rearing is a traditional practice in Nepal, and cattle rank first among all large ruminants in the country at 7.2 million heads [14]. Moreover, the dairy industry has developed rapidly in the last decades [15], but reproductive diseases such as leptospirosis in cattle have yet to be well assessed. The economic impact of leptospirosis on cattle production and its negative consequences on the livelihood of the poor and marginal farmers are underestimated [16].

Leptospirosis is one of Nepal’s top six priority zoonoses [17]. However, adequate surveillance and reporting are still lacking in Nepal [18]. After the first report of human leptospirosis in 1981, hundreds of cases with anti-*Leptospira* antibodies were reported among human and animal populations [18–20]. The seroprevalence of leptospirosis in human studies performed in different clinical settings varies from 2 to 28.7% [18, 19, 21, 22]. Some of these studies supported the idea that animal contact, particularly with cattle and buffaloes, was a likely source of infection in humans [18, 20].

In the context of Nepal, there is limited research and documentation on animal leptospirosis [23 - 26]. Existing studies have primarily focused on either canine or bovine populations and are limited to specific geographic regions. Among bovine species, previous studies have mainly investigated the prevalence of leptospirosis in mixed populations such as cattle, buffaloes, and yaks, often involving relatively small sample sizes. Commercial cattle farming contributes significantly to the country’s milk supply, with Rupandehi ranked among the top ten milk-producing districts in Nepal [27]. To the best of our knowledge, no study has identified and presented the risk factors associated with leptospirosis in the commercial cattle population of Nepal. Our study aimed to identify both the farm-level and animal-level risk factors that could contribute to the seropositivity of leptospirosis, specifically in cattle in the Rupandehi district of Nepal. Assessing these risk factors at farm levels could improve our understanding of leptospirosis as the priority zoonosis in the country.

## Materials and Methods

### Study design and study area

**Fig. 1:**
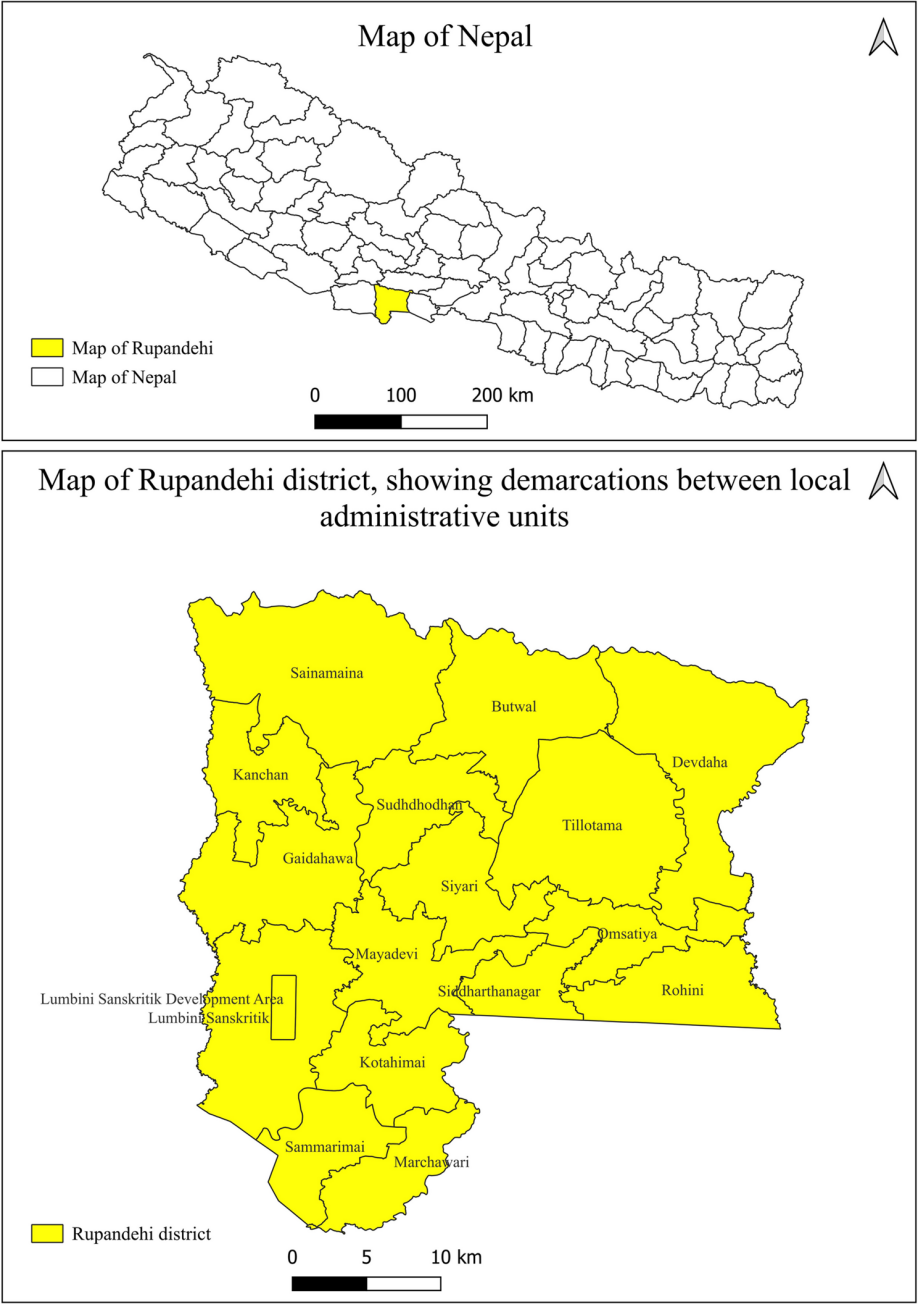
A map of Nepal with the study district, Rupandehi, highlighted at the top. Below is a detailed map of Rupandehi district showing the demarcation of local administrative levels

A cross-sectional study was conducted on commercial cattle farms of the Rupandehi district between March 2019 and April 2020 to estimate the seroprevalence and identify the risk factors associated with bovine leptospirosis. This study was conducted in the Rupandehi district in the southwestern region of Nepal **(**Fig. 1**)**. The district has tropical and subtropical climates with hot and humid weather in the summer months. The district shares the border with the neighboring nation India and has an international border quarantine where the importation of livestock exists between India and Nepal [28]. Rupandehi district is administratively divided into 16 local units, comprising one sub-metropolitan city, six urban municipalities, and ten rural municipalities.

### Study animals

In our study, animals were sampled from registered cattle dairy farms with sizes ranging from 5 to 200 animals, with a median herd size of 10. The majority of sampled animals were Holstein crossbreeds (61.31%), followed by Jersey crossbreeds (35.97%), and a small proportion of local breeds (2.72%). The animals’ ages ranged from 1 to 16 years, with a median age of 5 years. The median body condition score was 3 (range: 1–4). Most animals were female (97.82%), with a median parity of 2 (range: 0–8). Among female cattle, 16.16% were in early lactation, 15.88% in mid-lactation, 49.58% in late lactation, and 18.38% were non-lactating. Non-pregnant animals comprised the majority of the study population (83.84%), while 16.16% were pregnant.

### Sample size calculation and sampling techniques

The sample size was determined using open-source epidemiological software **“**OpenEpi” version 3.01 [29]. For selecting total farms, the expected prevalence was set as 50% with 5% desired precision at a 95% level of confidence that maximized the number of farm selections.

The formula for the above calculation **=** [DEFF*Np(1-p)] / [(d^2^/Z^2^_1−α/2_*(N-1) + p*(1-p)].

where DEFF = design effect, N = total number of animals, p = anticipated frequency, and d = absolute precision. The total number (N) of cattle farms to be selected by the above method was 206. To select 206 farms, we used a proportionate sampling procedure based on the number of farms at each local level. We utilized the following formula to identify the number of farms to be selected at each local level.

Number of farms selected at each local level =

1 $$\:\frac{Total\:number\:of\:farms\:in\:each\:local\:level}{Total\:number\:of\:farms\:in\:Rupandehi}$$

*sample size

The farms in each stratum were labeled ( 1…n_1_), and the required number of farms was chosen randomly using R (version 3.6.1) [30].

**Fig. 2:**
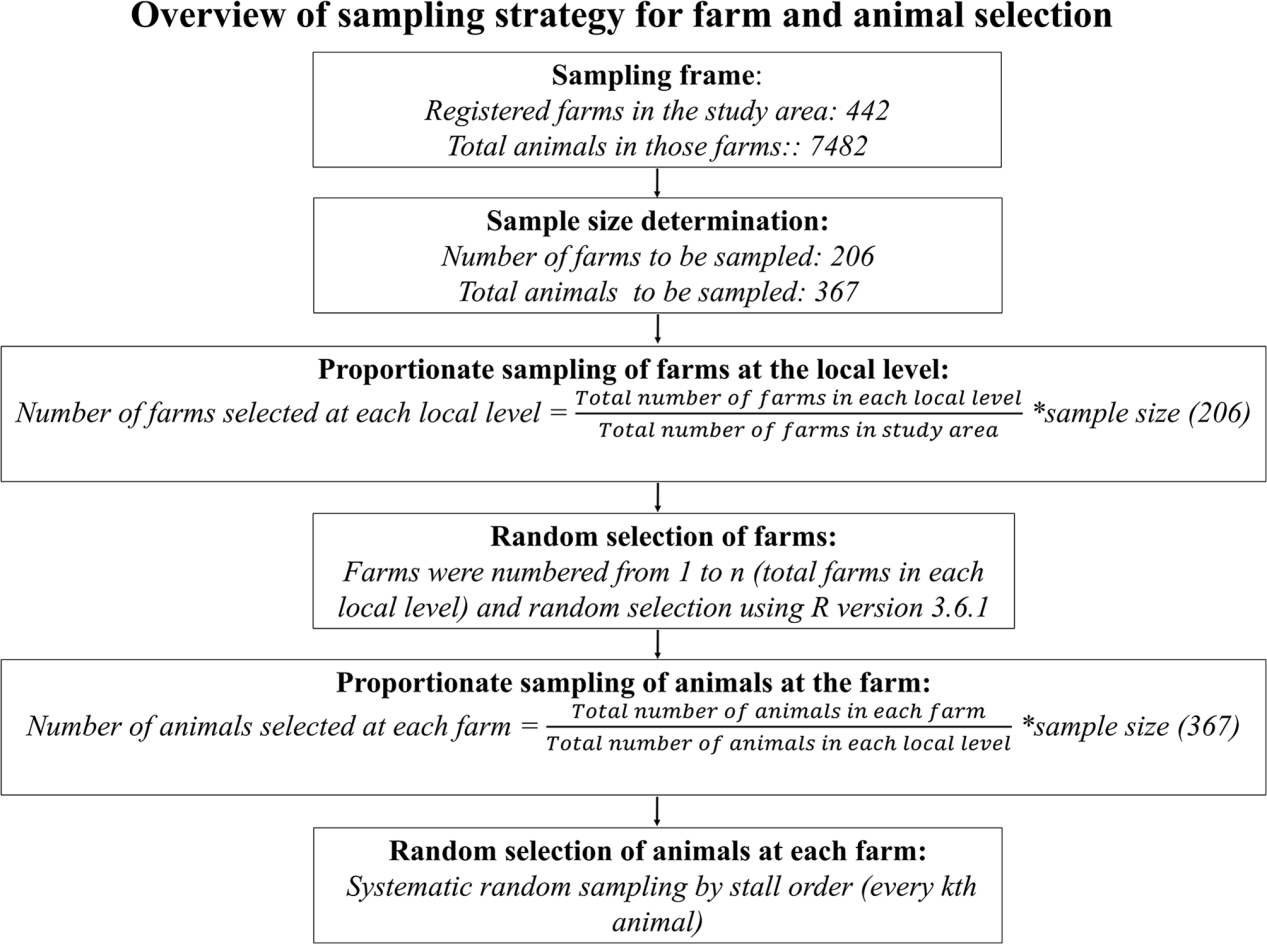
A flow diagram explaining the sampling procedures at different stages, including the selection of farms and individual animals using stratified and systematic random sampling methods in the study area

The sampling process consisted of two stages: firstly, farm-level sampling was done from 442 commercial farms located in all sixteen local levels (municipalities). Secondly, a proportionate number of animals were selected from each farm, giving a total individual sample size. For individual animal sampling, the same formula was used to calculate the total number of cattle to be sampled, keeping the animal population in the registered commercial farms at 7482 [31]. The total number of the individual animal (n) to be sampled by the above method was 367. The animals to be sampled at each local level were identified using a formula similar to the one mentioned above (for farms). We used systematic random sampling to select animals on each farm. The animals were tied in an orderly manner within the stall and were temporarily numbered from 1 to *n*. The sampling interval was calculated as *k = total number of animals on the farm / required number of animal samples from that farm*. A random starting number (*a*) was then selected between 1 and *k*, and every *k*-th animal starting from *a* was included in the sample. The summary of the sampling procedure is explained in a flow diagram in Fig. 2, and more details are provided in Supplementary Material **S2**. All samples and farm data were collected by two veterinarians, SP and SJ (also authors of this manuscript).

### Data and sample collection

#### Questionnaire data

The semi-structured questionnaire (Supplementary material, S1) was used to collect information on management status, biosecurity conditions, animal movement process of each farm, and owner’s knowledge of leptospirosis. The questionnaire was initially designed in English and later translated into Nepali. The questionnaire was pretested on ten farm owners from the study area as part of a pilot study. The coherence of responses with the questions and the clarity and ambiguity of the questionnaire were assessed to refine the survey questions and ensure the practical validity of the questionnaire for a full-phase study. Responses to the final questionnaire were obtained through in-person interviews with the owners of 206 farms at the time of blood sample collection. Written consent was obtained from the owners during the interview and sampling process.

#### Blood sample collection

A blood sample (5 ml) was collected from the jugular vein of each animal using sterile vacutainer tubes. The animals were properly restrained prior to sample collection, and trained final-year veterinary students performed the procedure to ensure efficiency and minimize stress. The blood collection tubes were properly labeled and transported to the laboratory the same day in an ice box to maintain the cold chain. The blood samples were allowed to remain overnight at room temperature to facilitate the serum separation, and the separated serum samples were transferred to 2 ml Eppendorf tubes. A total of 384 blood samples were collected. However, only 367 were retained in the study due to the lack of clear serum separation in the remaining samples. The collected serum samples were stored at -20˚C and transported to the Central Veterinary Laboratory (CVL), Kathmandu, for further analysis.

### Laboratory testing of samples

The indirect ELISA against leptospirosis antibody was performed using the “PrioCHECK^®^*L*. Hardjo Antibody ELISA” (Prionics Lelystad B.V., the Netherlands) kit. The process was performed according to the protocols provided within the kit (Ref no 7442080). The reading of the ELISA plates was performed by the ELISA reader (“Multiskan™ FC Microplate Photometer”) at an optical density (OD) of 450 nm within 15 min. The readings were interpreted using the protocol provided within the kit to determine the number of *L.* Hardjo seropositive samples. The estimated sensitivity (Se) and the specificity (Sp) of the indirect antibody ELISA were estimated to be (Se) 99% and (Sp) 85%), respectively [32–34].

### Test result validation and interpretation criteria

After the measurement of optical density, the corrected OD value was calculated by subtracting the OD value of reference (controls) and test sera (samples) with the mean OD value of the blanks (ELISA buffer). The percentage positivity (PP) of the reference and test sera were calculated as follows.

PP =

2 $$\:\frac{corrected\:OD\:\:of\:the\:test\:sample}{corrected\:\:OD\:\:of\:the\:\:positive\:control}*100$$

The result was validated if,

i. The mean OD of the blanks was < 0.150.
ii. The corrected OD of positive control was ≥ 1.000.
iii. The mean PP of negative control was < 20.
iv. The mean PP of weak positive control was between 20 and 60.

For each sample, the cut-off for percentage positivity for the test sera was determined as follows: PP < 20 was declared negative, PP between 20 and 45 was classified as inconclusive, and PP ≥ 45 was considered positive for *L.* Hardjo antibodies.

### Statistical analysis

First, the raw data was entered manually on MS excel^®^ spreadsheet from the paper-based questionnaire. After cleaning the data, the Excel file was converted to a CSV file and analyzed by R version 3.6.1 [30]. Farms with at least one sample positive were considered positive for *L.* Hardjo. The descriptive data analysis was performed to calculate the proportion of the seropositive farms. The continuous variables (farm size, age category, parity number) and an ordinal variable (body condition score) were converted to binary categorical variables using the quartiles (median as cut-off) to avoid the problem of linearity. Pearson’s Chi-square test was used to assess the associations between the categorical variables. Significantly associated and biologically plausible variables were retained in the model for further analysis.

A univariate and multivariable logistic regression analysis was performed to identify the farm-level risk factors. Similarly, due to a hierarchical structure in the data, animal-level risk factors were assessed via univariate and multivariable mixed-effect logistic regression analysis. Univariate analysis was performed to measure the association of each categorical variable with the outcome, “seropositivity to leptospirosis” (Table 2). The potential confounding effects of the variables were checked with the changes in the point estimates of the variables that remain in the multivariable model. Any changes in the coefficient with > 20% were considered confounders and were included in the final model.

We applied both manual and automated methods to select the model. For a manual method, variables with p-value ≤ 0.2 at the bivariate logistic regression analysis and those with biological plausibility were considered candidates for the final multivariable logistic regression model. Interaction effects between the variables in the final model were checked, and significant interaction (p-value < 0.05) was retained in the model. Stepwise selection and Akaike’s Information Criteria (AIC) were used for an automatic model selection method. The initial manually derived models were compared to the automated derived models, and both approaches ended up with the same set of variables for the final model. Any variables with a p-value < 0.05 at the final multivariable logistic regression analysis were considered risk factors.

The multicollinearity among the independent variables was checked for variance inflation factors (VIF) with the “vif” function in R, and variables with VIF coefficients > 10 were considered highly correlated. Independent variables detecting multicollinearity were removed from the model. The fitness of the final models was assessed by the Hosmer-Lemeshow goodness of fit function (hoslem.test) in R if they were fit.

For the animal-level analysis, the farm was considered a random effect to account for clustering at the farm level and other independent variables were chosen as fixed effects. We initially generated fixed-effects minimal base-line models and a base-line mixed model using the generalized linear mixed effect “glmer” function with a random intercept for farm ID. The AIC of the baseline model was compared to the AICs of several plausible models generated through a manual backward stepwise model selection procedure. We checked if the random effect was justified by comparing the AICs from the generalized linear model “glm” to AIC from the “glmer” model in R.

## Results

### Descriptive study on farm characteristics and farmers’ knowledge about leptospirosis

Of the 206 farms selected, the median farm size was 10, ranging from 1 to 200 cattle. The median age of the cattle on the farm was five years old. Of the 206 farms, the composition of homebred and purchased animals was 60.68% (125/206) and 39.32% (81/206) (Table 1). The majority, 93.69% (193/206), of the farms were based on a stall-feeding system, and only 6.31% (13/206) of them were taken for occasional grazing. About 85% (175/206) of the farms practiced an artificial insemination process for breeding their animals, and the remaining 15.04% (31/206) of the farms practiced the natural mating system.

We also asked the farmers about any additional jobs they had besides cattle farming. In our study, 32.5% (67/206) of the farmers used to work both in the rice field and cattle rearing. Only 18% (39/206) of the farmers had heard about zoonotic diseases. Interestingly, 17.48% (36/206) had a recent history of muscle pain and fever. However, none of the farmers had heard about leptospirosis and lacked information to prevent the transmission of that disease into their livestock.

**Table 1:** Descriptive summary of farm, animal, and farmer-level variables and associated leptospirosis prevalence

Determinants	Categories	Total number of animals examined	Leptospirosis		Prevalence (95%CI)
			Positive	Negative	
**Farm characteristics**					
Cattle origin	Home bred	81	1	80	1.23 (0.03–6.88)
	Purchased	125	9	116	7.20 (3.29–13.67)
Farm size (median = 10)	> 10	93	9	84	9.67 (4.44–18.37)
	<=10	113	1	112	0.89 (0.02–4.90)
Farm structure	Head to head	71	6	65	8.45 (3.1–18.39)
	Single	110	3	107	2.73 (0.56–7.97)
	Tail to tail	25	1	24	4.00 (0.1–22.29)
Bedding type	Mat	10	1	9	10.00 (0.25–55.72)
	None	52	3	49	5.77 (1.17–16.54)
	Saw dust	88	3	85	3.41 (0.7–9.96)
	Straw	56	3	53	5.36 (1.12–15.94)
Source of water	Tap	116	6	110	5.17 (1.9–11.26)
	Well	90	4	86	4.44 (1.21–11.38)
Feeding type	Occasional grazing	13	1	12	7.69 (0.19–42.86)
	Stall feeding	193	9	184	4.66 (2.13–8.85)
Breeding type	Artificial insemination	175	9	166	5.14 (2.35–9.76)
	Natural mating	31	1	30	3.23 (0.08–17.97)
Separate calving pen	No	180	9	171	5.00 (2.29–9.49)
	Yes	26	1	25	3.85 (0.1–21.43)
Common grazing ground	No	184	9	175	4.89 (2.24–9.29)
	Yes	22	1	21	4.55 (0.12–25.33)
Contact with another herd	No	23	0	23	NA*
	Yes	183	10	173	5.46 (2.62–10.05)
Contact with wild animals	No	156	6	150	3.85 (1.41–8.37)
	Yes	50	4	46	8.00 (2.18–20.48)
Grazing with pasture with community dog access	No	188	9	179	4.79 (2.19–9.09)
	Yes	18	1	17	5.56 (0.14–30.95)
Cat feces on the farm	No	134	5	129	3.73 (1.21–8.71)
	Yes	72	5	67	6.94 (2.25–16.21)
Presence of rodents on farm	No	67	1	66	1.49 (0.04–8.32)
	Yes	139	9	130	4.24 (1.71–8.74)
Feed contamination with the soil	No	79	2	77	2.53 (0.31–9.15)
	Yes	127	8	119	6.30 (2.72–12.41)
Knowledge about zoonosis	No	167	10	157	5.99 (2.87–11.01)
	Yes	39	0	39	NA*
**Owner’s knowledge and practice**					
Owner’s knowledge about leptospirosis	No	206	10	196	4.85 (2.33–8.93)
Owners working in rice fields and working at cattle farms	No	27	1	26	3.70 (0.09–20.64)
	Yes	179	9	170	5.03 (2.3–9.54)
Work with barefoot on rice field	No	58	1	57	1.72 (0.04–9.61)
	Yes	148	9	139	6.08 (2.78–11.54)
**Animal characteristics**					
Sex	Female	359	13	346	3.62 (1.93–6.19)
	Male	8	1	7	12.50 (0.32–69.65)
Age category (Median = 5)	≤ 5	242	11	231	4.55 (2.27–8.13)
	> 5	125	3	122	2.40 (0.49–7.01)
Breed category	Holstein cross	225	6	219	2.67 (0.98–5.80)
	Jersey cross	132	7	125	5.30 (2.13–10.93)
	Local	10	1	9	10.00 (0.25–55.72)
Body condition score (Median = 3)	≤ 3	357	13	344	3.64 (1.94–6.23)
	> 3	10	1	9	10.00 (0.25–55.72)
Parities number in females (Median = 2)	≤ 2	210	8	202	3.81 (1.64–7.51)
	> 2	149	5	144	3.36 (1.09–7.83)
Pregnancy status of female	Pregnant	58	2	56	3.45 (0.42–12.46)
	Not pregnant	301	11	290	3.65 (1.82–6.54)
Stage of Lactation	Non-lactating	66	6	60	9.09 (3.34–19.79)
	Early	58	3	55	5.17 (1.07–15.12)
	Mid	57	2	55	3.51 (0.42–12.67)
	Late	178	2	176	1.12 (0.14–4.06)
Abortion history	No	336	13	323	3.87 (2.06–6.62)
	Yes	24	0	24	NA*
History of repeat breeding	No	206	4	202	1.94 (0.53–4.97)
	Yes	153	9	144	5.88 (2.69–11.17)
History of retained placenta	No	336	13	323	3.87 (2.06–6.62)
	Yes	23	0	23	NA*

*NA = Not applicable (when there was no prevalence), CI: Confidence Interval

### Farm level and animal level Seroprevalence

In our study, samples from 206 farms were tested against leptospirosis. Out of them, 10 (4.85%; 95% CI: 2.35–8.75) farms had at least one seropositive animal. Likewise, 367 blood samples were collected from those 206 farms. In the 367 serum samples, 14 (3.81%; 95% CI: 2.1–6.3) tested positive for *L. Hardjo*.

### Univariate regression analysis for farm-level risk factors

A total of thirteen farm-level risk factors were assessed through univariate analysis (Table 2**)**. The purchased cattle farms had higher odds of detecting *L. Hardjo* (OR: 6.21, 95% CI: (0.77–49.96, p-value = 0.086) compared to the home-bred farms, but the finding was only borderline significant. The cattle farms with a farm size of > 10 were 12 times more likely to be seropositive compared to those with a farm size of ≤ 10 (OR: 12.00, 95% CI: 1.49–96.56, p-value = 0.02. All other factors were found non-significant with the farm level seropositivity.

**Table 2:** Farm-level risk factors associated with seropositivity for *Leptospira* Hardjo based on univariate logistic regression analysis. The outcome variable for this analysis was the farm status of *Leptospira* Hardjo (positive or negative)

Determinants	Categories	Crude OR	95% CI	*p*- value
**Farm Characteristics**				
Cattle origin	Home breed	Ref		
	Purchased	6.21	(0.77–49.96)	0.086
Farm size (median = 10)	<=10	Ref		
	> 10	12	(1.49–96.56)	0.020*
**Farm Management system**				
Farm structure	Single	Ref		
	Head to head	3.29	(0.80–13.62)	0.1
	Tail to tail	1.49	(0.15–14.91)	0.736
Bedding type	None	Ref		
	Mat	1.81	(0.17–19.46)	0.622
	Saw dust	0.58	(0.11–2.97)	0.51
	Straw	0.92	(0.18–4.80)	0.926
Source of water	Well	Ref		
	Tap	1.17	(0.32–4.29)	0.81
Feeding system	Stall feeding	Ref		
	Occasional grazing	1.7	(0.20–14.58)	0.627
Breeding type	Natural Breeding	Ref		
	Artificial insemination	1.63	(0.20–13.31)	0.65
Cat feces seen around farms	No	Ref		
	Yes	1.93	(0.54–6.89)	0.314
Feed contamination with the soil	No	Ref		
	Yes	2.59	(0.54–12.51)	0.237
Rodents seen around farms	No	Ref		
	Yes	4.57	(0.57–36.84)	0.154
Owners worked on rice fields with bare feet	No	Ref		
	Yes	3.7	(0.46–29.81)	0.22
Contact with wild animals	No	Ref		
	Yes	2.17	(0.59–8.04)	0.244
Separate calving pen	No	Ref		
	Yes	0.76	(0.09–6.26)	0.799

CI: Confidence Interval, OR: Odds Ratio, *p-value < 0.05

### Multivariable logistic regression analysis for farm-level risk factors

In the final farm-level multivariable model, the farm-level risk factor “cattle origin” was marginally associated with seropositivity to leptospirosis, while “farm size” showed a statistically significant association (Table 3**)**. The purchased cattle farms had higher odds of detecting leptospirosis (OR: 7.25, 95% CI: 0.88–59.46, p-value = 0.065) than home-bred cattle farms. Cattle farms with a farm size of > 10 animals were about 13 times more likely to be seropositive for leptospirosis (OR: 13.34, 95% CI: 1.64-108.42, p-value = 0.015) than those with a farm size of ≤ 10 animals.

**Table 3:** Farm-level risk factors associated with leptospirosis infection on commercial dairy farms by multivariable logistic regression analysis. The outcome variable for this analysis was the farm status of *Leptospira* Hardjo (positive or negative)

Determinants	Factor level	Adjusted OR	95%CI	*p*-value
Cattle origin	Home bred		Ref	
	Purchased	7.25	(0.88–59.46)	0.065
Farm size	≤ 10		Ref	
	> 10	13.34	(1.64-108.42)	0.015*

CI: Confidence Interval, OR: Odds Ratio, *p-value < 0.05

### Univariate and multivariable mixed effect binomial logistic regression models

For the animal level univariate mixed effect binomial logistic regression model analysis, sex, body condition score, parity number, common grazing field and grazing in pasture with the community dog access, history of repeat breeding, provision of grazing field, and status of community dog access to grazing field were considered in the model (Table 4**)**. Only pasture with community dog access was marginally associated (p-value = 0.05) with the outcome variable (disease). The fixed effect estimate for grazing cattle in pastures with community dog access was marginally significant (OR:13.3 (SE = 6.8, *p* = 0.051), indicating a positive association testing positive for leptospirosis but the result is not statistically significant.

For the multivariable mixed effect binomial logistic regression model, predictors with p-value ≤ 0.2 in the univariate mixed effect model were considered. None of the predictors were statistically significant (p-value > 0.05). Hence, the result of the multivariable mixed effect model was not further discussed or reported here.

**Table 4:** Animal-level risk factors associated with cattle *L. Hardjo* Seroprevalence in rupandehi, by univariate mixed effect binomial logistic regression models. The outcome variable for this analysis was the animal status of *Leptospira* Hardjo (positive or negative)

Determinant	Categories	Estimates	Standard error (SE)	*p*-value
**Animal Characteristics**				
Sex	Female	Ref		
	Male	10.17	6.87	0.14
Body condition score (Median = 3)	<=3	Ref		
	> 3	10.11	6.74	0.13
Parity number in cow (Median = 2)	<=2	Ref		
	> 2	3.23	2.27	0.15
History of repeat breeding	No	Ref		
	Yes	1.28	0.92	0.16
**Animal movements and biosecurity**				
Common grazing field	No	Ref		
	Yes	2.07	1.42	0.14
Grazing in pasture with community dog access	No	Ref		
	Yes	13.28	6.80	0.05^b^

^b^ Marginally significant

## Discussion

Given Nepal’s diverse agroecological zones, mixed farming practices, frequent human-animal contact, low levels of farm biosecurity, and an open border with India, there is a clear need for a better understanding of the epidemiology of leptospirosis. The status of leptospirosis in dairy cattle in Nepal remains largely unknown due to poor disease knowledge, limited laboratory capacity, little scientific research, and inadequate disease surveillance [35]. In this context, our study aimed to address this gap by estimating the prevalence of leptospirosis, identifying its risk factors, and assessing farmers’ knowledge about the disease. The findings provide a valuable baseline for researchers and policymakers to better understand the status of leptospirosis in the cattle population and to inform effective future interventions for its control.

We estimated the farm level and animal level seroprevalence of leptospirosis as 4.85% (10/206) and 3.81% (14/367), respectively, among the cattle farms in the Rupandehi district of Nepal. The prevalence of bovine leptospirosis can vary significantly depending on seasonal patterns, geographic location, and a range of environmental and management-related factors [36]. A study conducted by Khanal et al. highlighted the seasonal variation in disease prevalence in the cattle population in Nepal, with a prevalence of 4.51% in post-monsoon and 6% in the pre-monsoon season [24]. Also, the literature suggests that disease prevalence varies greatly in neighboring country India, with seroprevalence ranging from 1.2% [37] to 87% [38] in cattle population, and higher prevalences when multiple serovars are tested.

The farms that regularly purchase animals from other farms were more likely to detect leptospirosis than the homebred farms. It is epidemiologically justifiable that the farms that have higher movements of animals during the animal trade are more likely to contract the disease [40–42]. The Rupandehi district of Nepal, located at a key trade transit point with India and neighboring provinces, sees a higher exposure to risks for cattle farms that are transported between destinations compared to those that are confined. Our findings showed that the cattle farms of > 10 animals had higher odds of reporting the disease compared to farms with animals < 10. The bigger farm size is a risk factor for leptospirosis, and many studies have already proven it [34, 42]. The high stocking density in the bigger farm size farms had a higher risk of getting the diseases due to the frequent selling or purchasing of animals, and the contact between infectious and susceptible animals occurs more often [41].

Our findings showed that the cattle taken in the pasture with community dog access were borderline significant for the seropositivity of leptospirosis at the univariate mixed effect regression logistic regression model. The role of dogs as reservoirs or sentinels of environmental contamination has been documented in the past [43, 44]. Nepal has a large population of community dogs in the cities and village areas. These dogs frequently move in search of food and defecate openly in the pasture, which leads to the contamination of grazing ground. These dogs are a potential source of leptospirosis for both farmed animals and humans. A study conducted in Brazil [7] demonstrated that the presence of dogs in the pasture was associated with detecting *Leptospira* spp. antibodies in cattle. Nepal’s central, provincial, and local governments need to formulate sustainable plans to manage the population of community dogs.

Noteworthily, only a few of the farmers had prior knowledge of zoonotic diseases, and none were aware of leptospirosis. While some reported that their cattle occasionally experienced fever or abortion, they did not recognize these signs as potentially indicative of leptospiral infection. This lack of awareness poses a serious public health concern and creates a major barrier to controlling the transmission of the disease across human, animal, and environmental interfaces. Although the Government of Nepal has identified leptospirosis as one of its six priority zoonotic diseases [17], our findings show that public health education and outreach efforts have been inadequate. Awareness and risk communication strategies should specifically target high-risk populations, such as livestock farmers, animal health workers, and individuals living in flood-prone or high-rainfall areas where the risk of environmental exposure to *Leptospira* spp. is elevated. In addition, surveillance systems should be strengthened, laboratory diagnostic capacity should be expanded, and case reporting mechanisms should be improved to support early detection, accurate risk assessment, and effective implementation of control measures. Given the ecological complexity of leptospirosis, the involvement of multiple animal reservoirs, and the environmental persistence of the pathogen, adopting a One Health approach is essential. This requires coordinated collaboration among public health professionals, physicians, veterinarians, environmental health specialists, and policymakers. These stakeholders should work together to develop evidence-based policies, enhance health system preparedness, and implement practical interventions that reduce transmission risks and disease burden in both human and animal populations in the country.

This study also has some limitations worth noting. We might have missed other important serovars of leptospirosis other than *L.* Hardjo and may have underestimated the seroprevalence in cattle due to the availability of a sole detection kit. Some cattle farm owners did not remain on the farms, and the information was obtained from the less educated farm workers, and their level of understanding may not represent the idea of all cattle owners in general. Potential recall bias may have occurred when collecting information about the animals, as most farmers had not maintained proper records on their farms. Additionally, the research was conducted in one district of the Terai region of Nepal, and results from our study might differ from the districts of the Hilly and Mountain regions of Nepal. It would also be valuable to assess the leptospirosis status of farmers from leptospirosis-positive farms; however, our study was limited to dairy cattle and did not include human subjects. Overall, all these limitations should be considered when interpreting the results, and future studies could address these constraints for a more comprehensive understanding of leptospirosis prevalence in cattle in Nepal.

## Conclusions

This study provides critical insights into farm- and animal-level risk factors for leptospirosis in dairy cattle in Nepal. Key risk factors, such as frequent cattle purchases and larger farm sizes, underscore the influence of animal movement and herd scale on disease transmission dynamics. Although only marginally significant, our findings also suggest a potential role of community dogs in transmitting disease to cattle. Given the zoonotic nature and ecological complexity of the disease, which involves multiple host species, a One Health approach is essential for effective control and prevention in both human and animal populations. Collaboration among stakeholders is required to design evidence-based policies, improve health system preparation, and implement practical interventions that minimize transmission risks and the overall disease burden across the country. We recommend expanding research to a nationwide scale and implementing control measures through a One Health framework to reduce the incidence of zoonotic diseases such as leptospirosis in Nepal.

## Electronic supplementary material

Below is the link to the electronic supplementary material.

Supplementary Material 1: **S1**: Questionnaire

Supplementary Material 2: **S2**: Sampling frame

## Data Availability

The data used in this study can be available from the corresponding authors upon reasonable request.

## Abbreviations

GDP: Gross Domestic Product
ELISA: Enzyme Linked Immunosorbent Assay
WHO: World Health Organization
OD: Optical Density
CVL: Central Veterinary Laboratory
NVC: Nepal Veterinary Council
PP: Percentage Positivity
