# Supplementary material for: Seroprevalence and risk factors associated with Leptospira Hardjo among commercial dairy cattle farms of Rupandehi district, Nepal
Source: BMC Vet Res. 2025 Jul 5;21:442. doi: 10.1186/s12917-025-04882-x (PMC12228320; doi:10.1186/s12917-025-04882-x)
Supplement: Supplementary file 1 — Supplementary Material 1: S1: Questionnaire [file 12917_2025_4882_MOESM1_ESM.docx]

**QUESTIONNAIRE**

Serial no: ………………… Date: ………………………

**FARM INFORMATION:**

| Farm No. |  |
| --- | --- |
| Name of the farm/ owner |  |
| Municipality/VDC |  |
| Ward No. |  |
| Locality |  |
| Herd size |  |
| Animals other than cattle on farms | a. Buffalo b. Goat c. Sheep d. Dog e. Cat f. Chickens g. Others (…………) |
| Contact No. |  |

| S.N. | Animal No. | Age | Sex | Breed | Body Condition  Score  (1-5) | Parity no | Pregnancy state (Y/N) | Lactation state (Y/N) | History of abortion/  stillbirths  (Y/N) | History of Repeat breeding  (Y/N) | Tick infestation(Y/N) |
| --- | --- | --- | --- | --- | --- | --- | --- | --- | --- | --- | --- |
|  |  |  |  |  |  |  |  |  |  |  |  |
|  |  |  |  |  |  |  |  |  |  |  |  |
|  |  |  |  |  |  |  |  |  |  |  |  |

**INDIVIDUAL ANIMAL LEVEL INFORMATION:**

**Management Aspect:**

1. What is the origin of cattle? a. Home breed b. Purchased c. Donation. d. Both a and b
2. What kind of housing system do you have on your farm?

a. Head-to-head b. Tail- to- tail c. Single d. Free

1. What is the type of bedding you use for your cattle? a. Straw b. Saw dust c. Mat d. None
2. What is the source of water for animals? a. Tap b. Well c. Pond d. River e. Others
3. How do you feed your cattle? a. Stall feeding b. Full time grazing c. Stall feeding and occasional grazing
4. How do you breed your cattle? a. Natural mating b. Artificial Insemination
5. Do you have separate calving pens? a. Yes b. No
6. Do animals come in contact with other domestic animals on the farm? a. Yes b. No
7. If yes, which animals are likely to get contact? ……………………….
8. Do you use common grazing fields for cattle and other domestic species? a. Yes b. No
9. Do animals come in contact with animals of another herd? a. Yes b. No
10. Do farm animals come in contact with wild animals? a. Yes b. No
11. Do you graze your cattle in pastureland where community dogs have access? a. Yes b. No
12. Have you seen cat feces around the farm? a. Yes b. No
13. Have you encountered a rat/mouse in the animal shed? a. Yes b. No
14. Does the feed have a chance of getting contaminated with the soil? a. Yes b. No

**Owner’s information**

1. Have you heard about zoonotic diseases? a. Yes b. No
2. Have you heard about Leptospirosis? a. Yes b. No
3. Do you know that leptospirosis can be transmitted from animals to humans? a. Yes b. No
4. Do you take your cattle to rice fields for grazing after the rice is reaped? a. Yes b. No
5. Do you work in muddy rice fields? a. Yes b. No
6. Do you work barefoot in rice fields during summer? a. Yes b. No

**Owner’s consent**

I agree to disclose the information collected to a wider audience, keeping our personal information anonymous.

a. Name of owner/farmworker…………………….

b. Signature of owner/farmworker …………………………………
