# Supplementary material for: Seroprevalence and risk factors associated with Leptospira Hardjo among commercial dairy cattle farms of Rupandehi district, Nepal
Source: BMC Vet Res. 2025 Jul 5;21:442. doi: 10.1186/s12917-025-04882-x (PMC12228320; doi:10.1186/s12917-025-04882-x)
Supplement: Supplementary file 2 — Supplementary Material 2: S2: Sampling frame [file 12917_2025_4882_MOESM2_ESM.docx]

| Strata number | Municipality (local level) | Total no. of farms | Number of selected farms | Total no. of animals | Number of  Selected animals |
| --- | --- | --- | --- | --- | --- |
|  |  |  |  |  |  |
| 1 | Tilottama municipality | 105 | 49 | 1754 | 86 |
| 2 | Sainamaina municipality | 45 | 21 | 810 | 40 |
| 3 | Butwal sub-metropolitan municipality | 41 | 19 | 675 | 33 |
| 4 | Devdaha municipality | 83 | 39 | 1323 | 65 |
| 5 | Suddhodhan rural municipality | 53 | 25 | 742 | 36 |
| 6 | Kanchan rural municipality | 15 | 7 | 361 | 18 |
| 7 | Gaidahawa rural municipality | 9 | 4 | 136 | 7 |
| 8 | Lumbini Sanskrit municipality | 7 | 3 | 146 | 7 |
| 9 | Sammarimai municipality | 11 | 5 | 176 | 9 |
| 10 | Marchawari rural municipality | 0 | 0 | 0 | 0 |
| 11 | Kotahimai rural municipality | 1 | 0 | 11 | 0 |
| 12 | Siddharthanagar municipality | 8 | 4 | 119 | 6 |
| 13 | Rohini rural municipality | 4 | 2 | 40 | 2 |
| 14 | Om satiya rural municipality | 12 | 5 | 239 | 12 |
| 15 | Mayadevi rural municipality | 6 | 3 | 106 | 5 |
| 16 | Siyari rural municipality | 42 | 19 | 844 | 41 |
|  | Total | 442 | 206 | 7482 | 367 |

S2: The number of farms and animals chosen using a proportionate sampling technique in Rupandehi district, Nepal.

Total farms/animals selected in each local level = $\frac{Total number of farms/animals in each local level}{Total number of farms/animals in Rupandehi}$ *estimated sample size
